# The Lyme and Tickborne Disease Dashboard: A map-based resource to promote public health awareness and research collaboration

**DOI:** 10.1371/journal.pone.0260122

**Published:** 2021-12-01

**Authors:** Frank C. Curriero, Cara Wychgram, Alison W. Rebman, Anne E. Corrigan, Anton Kvit, Timothy Shields, John N. Aucott

**Affiliations:** 1 Department of Epidemiology, Spatial Science for Public Health Center, Johns Hopkins Bloomberg School of Public Health, Baltimore, Maryland, United States of America; 2 Division of Rheumatology, Department of Medicine, Lyme Disease Research Center, Johns Hopkins University School of Medicine, Baltimore, Maryland, United States of America; University of Kentucky College of Medicine, UNITED STATES

## Abstract

With the incidence of Lyme and other tickborne diseases on the rise in the US and globally, there is a critical need for data-driven tools that communicate the magnitude of this problem and help guide public health responses. We present the Johns Hopkins Lyme and Tickborne Disease Dashboard (https://www.hopkinslymetracker.org/), a new tool that harnesses the power of geography to raise awareness and fuel research and scientific collaboration. The dashboard is unique in applying a geographic lens to tickborne diseases, aiming not only to become a global tracker of tickborne diseases but also to contextualize their complicated geography with a comprehensive set of maps and spatial data sets representing a One Health approach. We share our experience designing and implementing the dashboard, describe the main features, and discuss current limitations and future directions.

## Introduction

Lyme disease is a vectorborne disease transmitted through the bite of infected *Ixodes* ticks which are commonly found throughout temperate regions of North America, Europe, and Asia. Since *Borrelia burgdorferi* was recognized as the etiological agent of Lyme disease 40 years ago, the number of reported cases and their geographic distribution have increased. Due to changes in climate, land use patterns, and the distribution of reservoir hosts, *Ixodes* tick populations have increased and expanded to new geographic areas [1, 2]. Although the global burden of Lyme disease is difficult to estimate, it is thought to be underrepresented by human disease surveillance data routinely collected by governmental organizations. Lyme disease is the most reported vectorborne disease in the US, with approximately 35,000 cases reported through passive surveillance each year. However, according to a study by the US Centers for Disease Control and Prevention (CDC), insurance records indicate that the actual clinical burden of Americans treated for Lyme disease is closer to half a million [3]. Underreporting, especially at a rate of more than 90 percent, is a concern because effective public health responses require accurate and up-to-date information on disease spread and risk. Moreover, under-recognition of this epidemic makes it difficult for Lyme disease and other emerging tickborne threats to galvanize the level of research interest and funding that is needed for improved surveillance, prevention, diagnosis, and treatment.

In the spring of 2020, the Johns Hopkins Lyme Disease Working Group expressed interest in a mapping initiative for tickborne diseases. At the time, the Johns Hopkins COVID-19 dashboard had become the leading resource for mapping the spread of COVID-19. Its popularity magnified the value of online dashboards for communicating public health information through data visualization. The working group envisioned a dashboard that similarly could become a resource and a point of reference in the tickborne disease domain. Work on the dashboard began in the fall of 2020, bringing together a team from the Johns Hopkins Spatial Science for Public Health Center (https://www.jhsph.edu/research/centers-and-institutes/spatial-science-center-for-public-health) and the Johns Hopkins Lyme Disease Research Center (https://www.hopkinslyme.org). Our guiding mission statement is articulated in Table 1.

**Table 1:** Mission statement of the Johns Hopkins Lyme and Tickborne disease dashboard.

Harnessing the Power of Geography in Tickborne Disease Research
Lyme and other tickborne diseases are on the rise. More people are getting sick, ticks are spreading to new geographic locations, and new tickborne pathogens and diseases are being discovered. Our mission is to address this growing public health threat through a geographic lens. Our interactive mapping dashboard brings together disease, environmental, social, and other geographic data and makes them available to visualize and download. Through our dashboard, we aim to track and contextualize the impact of tickborne diseases while also highlighting data limitations and gaps in knowledge.
Our initiative is guided by a comprehensive One Health approach that utilizes multi-sectoral and multi-disciplinary collaboration to understand how the interdependence of humans, animals, and our shared environment impacts tickborne diseases. With this approach, we will advance understanding of the geography of tickborne diseases, improve awareness across multiple stakeholders, and motivate and support future research collaborations and communication.

## Methods

Our approach was to design the dashboard with researchers in mind while also making it accessible and useful to public health practitioners, policymakers, advocacy groups, and the general public. We also wanted the dashboard, as a mapping tool, to communicate to users why geography matters. We began by reviewing the literature on tickborne diseases and found that their geography is driven by a complex web of factors affecting host-vector-pathogen interactions and is not fully understood. The socio-ecological context in which disease risk varies geographically encompasses environmental factors such as land use, climate, topography, and vegetation, as well as demographic factors such as urban/rural residence, income, and education [4]. Tickborne diseases should be approached from a One Health perspective because they represent the interdependence of human, animal (both wild and domesticated), and environmental health, and our understanding and management of them requires the expertise and cooperation of physicians, veterinarians, ecologists, and other stakeholders. We saw from our early research the potential for our dashboard to become not simply a mapping tool for displaying human tickborne disease data, but a one-stop destination for relevant geographic data. In reviewing existing mapping and data visualization resources related to tickborne diseases, we found that most are geographically limited tick and/or human case surveillance resources operated by universities or local health departments. Moreover, none brings together various geographic data in a single place in a way that supports One Health-oriented research and collaboration.

With the one-stop destination approach in mind, we then identified sources of mappable data. Because we planned to launch the dashboard in time for Lyme Disease Awareness Month in May of 2021, we decided to use our limited time to focus on the US and Canada for the initial launch. We gathered human tickborne disease data for the US from CDC, which publishes county-level data for Lyme disease and state-level data for other nationally notifiable tickborne diseases [5, 6]. Although the Public Health Agency of Canada (PHAC) does not publish tickborne disease data sets for the entire country, we were able to combine some limited Lyme disease data from PHAC with data from provincial public health organizations [7–14]. The smallest geographic scale at which these data are publicly available is the health region (also called unit or zone, depending on the province), which is a grouping of census divisions. We gathered environmental data from the US Geological Survey [15, 16], the National Oceanic and Atmospheric Administration [17], the US Department of Agriculture [18], Natural Resources Canada [19, 20], Statistics Canada [21], Environment and Climate Change Canada [22], and Agriculture and Agri-Food Canada [23]. We obtained socio-demographic data primarily from the respective census programs [24, 25]. For the US, we identified two additional categories and sources of data: tick surveillance data from CDC [26] and search engine query data from Google Trends [27]. In general, we selected data that either already were available at the county level (or census division level for Canada) or that we could process to such a level in a geographic information system (GIS). We selected this level not only to maintain consistency with our mapping but also to facilitate ecological (or area-level) analyses, which are common in tickborne disease research due to the aggregation of human disease data to administrative geographies. We obtained geographic boundary shapefiles from the US Census Bureau, Esri, and Statistics Canada [28–31]. We processed the data in R 4.0.3 [32] and/or ArcGIS Pro 2.4.0 [33] and stored each map layer as a shapefile.

During the process of mapping our data, we began developing a dashboard prototype in R Shiny [34], an open-source R package that combines the analytical power of the R programming language with an interactive web application framework. We chose R Shiny because, compared to more out-of-the-box tools such as Tableau and ArcGIS Dashboards, R Shiny offered greater flexibility to control and customize the dashboard’s appearance and functionality. Moreover, we were already familiar with R and its spatial analysis capabilities, including its more advanced statistical capabilities (e.g., regression and cluster analysis) that we may consider incorporating in future versions of the dashboard. Although our primary focus with the prototype was to test and iterate the mapping functionality, we ultimately envisioned the dashboard as not simply a mapping tool but a full-fledged website that seamlessly integrates data visualization and descriptive information about tickborne diseases. Over several months, we improved the prototype based on internal discussions and feedback from several individuals representing scientists, clinicians, the government, as well as patients and patient advocates.

The next steps were to build out the website and optimize the R Shiny elements to support multiple simultaneous users. In the spring of 2021, we partnered with Appsilon, a data science company with expertise in R Shiny dashboards and website design. The most logical design approach was to embed the R Shiny elements within a static website (i.e., a website with fixed content and no server-side data processing) and host these elements on a separate and more powerful server (shinyapps.io), thereby allowing the dashboard to remain as fast as possible while some users perform more resource-intensive mapping operations.

## Results

The first version of the dashboard launched on May 3, 2021 and is accessible at https://www.hopkinslymetracker.org/. In this section, we highlight three features and discuss how they support the mission of the dashboard. The first two features comprise the two R Shiny mapping applications that are embedded within the website. The first application (labeled “Lyme Map” on the website) provides a data overview of Lyme disease and the second application (labeled “Data Explorer”) allows users to explore various tickborne disease-related maps. The third feature (labeled “Geography, Ticks and You”) is a standalone “storymap” about tickborne diseases that also serves to contextualize the data presented in the two mapping applications.

### Feature 1: “Lyme Map” application

**Fig 1:**
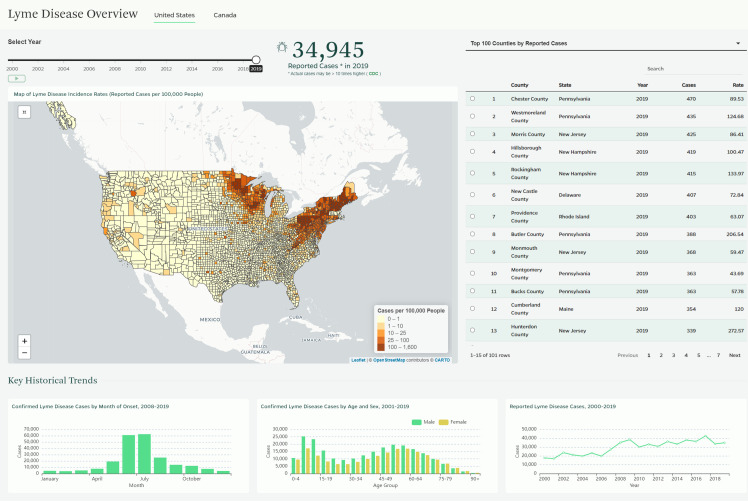
US view of “Lyme Map” application. The data in this figure are from CDC and the US Census Bureau and are in the public domain. Map tiles are reprinted under a CC BY license, with permission from CARTO, original copyright 2021. CARTO map tiles contain information from OpenStreetMap and OpenStreetMap Foundation, which is made available under the Open Database License.

The first application (Fig 1) resembles a traditional dashboard in that it provides a combined view of multiple metrics on a single platform. It has several structural components, including a map of Lyme disease incidence rates (i.e., reported cases per 100,000 people) in the US and Canada, a sliding control for the reporting year, and a table that ranks locations both by their absolute number of cases and their incidence rates. Epidemiologists use both case numbers and incidence rates to describe a disease in a population, although incidence rates tend to be more useful for comparing levels of a disease in locations with different population sizes.

The data visualized in this application draw from CDC, PHAC, and Canadian provincial public health organizations. The map allows users to compare locations and visualize how the geographic distribution of cases has changed over time. There is also a section that presents non-geographic trends, including the distribution of cases by month of disease onset and the distribution of cases by age group and sex. This first application provides a quick, by-the-numbers look at Lyme disease and is designed for a general audience interested in understanding where the disease is commonly reported. The US view improves upon the user experience design of CDC’s website, which spreads visualizations across multiple pages, has limited interactivity, and features an overly simplistic map of “high-” and “low-” incidence states. Our application’s interactive county-level map makes it clearer that incidence can vary substantially within the same state, and that localized hot spots can exist in otherwise low-incidence states. Although PHAC maintains a map of known risk areas for Lyme disease, and some Canadian provinces have infectious disease dashboards with Lyme disease maps and data, the Canadian view on our application is, to our knowledge, the only interactive map showing geographic variation in reported incidence rates for the entire country.

### Feature 2: “Data Explorer” application

**Fig 2:**
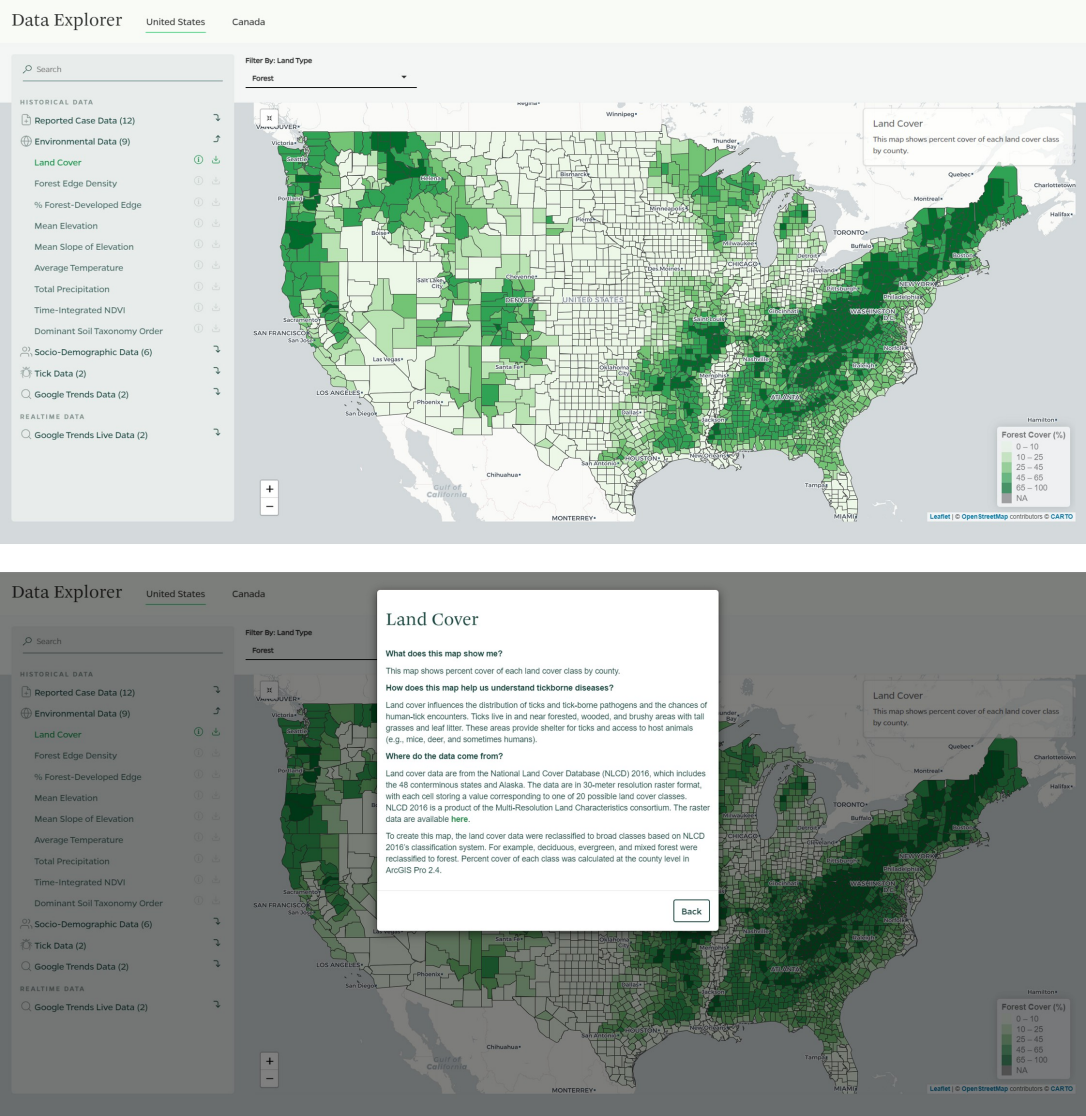
**A.** Land cover map with filtering controls on “Data Explorer” application. The data in this figure are derived from public domain data from the US Geological Survey. Map tiles are reprinted under a CC BY license, with permission from CARTO, original copyright 2021. CARTO map tiles contain information from OpenStreetMap and OpenStreetMap Foundation, which is made available under the Open Database License. **B.** Land cover map with popup window on “Data Explorer” application. The data in this figure are derived from public domain data from the US Geological Survey. Map tiles are reprinted under a CC BY license, with permission from CARTO, original copyright 2021. CARTO map tiles contain information from OpenStreetMap and OpenStreetMap Foundation, which is made available under the Open Database License.

The second application is a one-stop destination for geographic data, allowing users to toggle between various maps within the same application. The Reported Case Data category pulls together data from CDC reports so that users can easily visualize and compare the geographic distributions of different tickborne diseases. As we note in the Methods section, other data that may support understanding of tickborne disease geography often are spread across multiple government agency websites. Moreover, they are not always in a format that is accessible to those without GIS experience. For example, many environmental data derived from satellite imagery, such as land cover and vegetation, come in raster format (i.e., composed of pixels) and are more meaningful when summarized at a more operational geographic scale, such as counties. A key feature of our application is that relevant data are available in one place and any necessary data processing has been performed such that users can, with a few exceptions, visualize all the map layers on the same geographic scale. A second feature that speaks to our one-stop destination approach is that the underlying data (in both CSV and GIS shapefile format) and metadata for each map are immediately available to download from the application as a .zip file, enabling users to explore the data sets further and use them in their decision-making and research. A third feature is the application’s interactivity. Users can select the data that they want to display on the map and interact with certain maps that have filtering controls. For example, like the “Lyme Map” application, all the maps under Reported Case Data can be filtered by reporting year. Under Environmental Data, the Average Temperature map can be filtered by year and season and the Land Cover map (Fig 2A) can be filtered by land cover type. Each map layer has a clickable popup window (Fig 2B) that explains what the map shows, why the map is relevant to tickborne diseases, the source of the data, and, if applicable, how we processed the data. The popup windows are an interactive feature that supports user interpretation and understanding by contextualizing each map’s relevance to tickborne diseases.

### Feature 3: “Geography, Ticks and You” storymap

**Fig 3:**
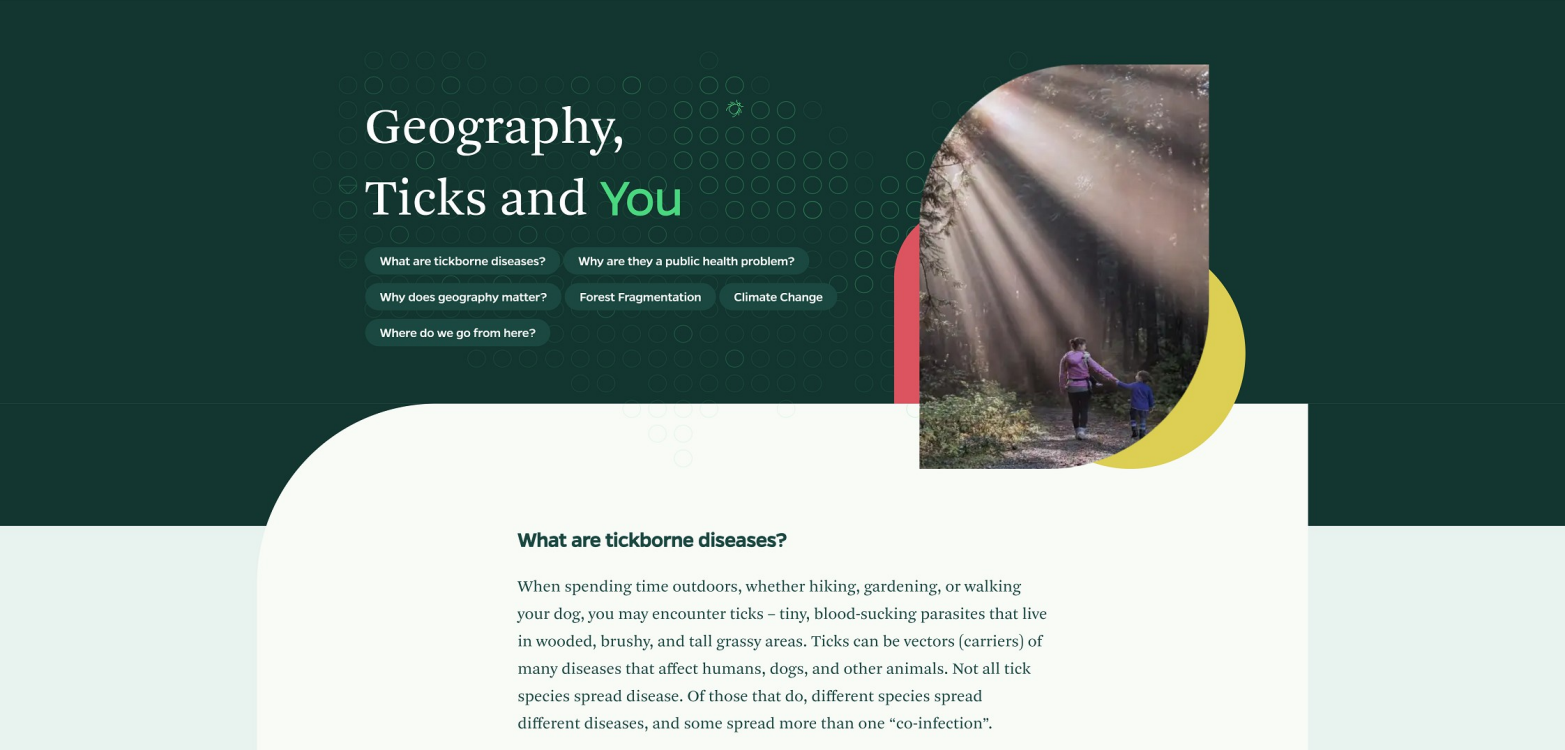
Navigation menu on the “Geography, Ticks and You” storymap.

Because maps by themselves can lack context and personal relevance, the dashboard also features a “storymap” component (Fig 3) that helps connect readers, especially lay readers, to the problem of tickborne diseases. The storymap feature integrates maps, data, text, and images in order to tell the story of why tickborne diseases are a growing public health problem and how aspects of geography can affect disease risk. Drawing from our literature review, we discuss how ticks and tick-host relationships depend on environmental conditions and how climate change and land use change may be driving the growth and spread of tick populations. We also highlight gaps in our understanding of the spatial epidemiology of tickborne diseases and communicate the need for increased prioritization, funding, and research.

## Discussion

Together, the three highlighted features illustrate the uniqueness of our approach and the potential of the dashboard to educate the public, support research, and motivate action. By bringing together data and information on disease epidemiology, ticks, the environment, and population demographics, we advance a One Health approach to tickborne diseases. A One Health approach recognizes that human, animal, and environmental health are inherently linked and advocates for multisectoral and multidisciplinary collaboration to achieve a broad range of prevention and control activities, whether they are directed at public and institutional awareness, surveillance (human, veterinary, and tick), or management and protection of wildlife and natural resources.

### Disease data challenges

One of the lessons from the Johns Hopkins COVID-19 dashboard is that disease tracking tools are most useful when they are constantly updated with the most current and accurate data available. To that end, our tickborne disease mapping initiative was constrained by several factors. In the US, publicly available tickborne disease data are limited to reports published by CDC and individual states. Participation in CDC’s National Notifiable Diseases Surveillance System (NNDSS) is voluntary on the part of local and state public health departments. NNDSS is a passive surveillance system that relies on health care providers reporting cases. Lyme disease reporting can be burdensome because positive laboratory reports do not include the exposure and clinical information required to classify cases according to CDC’s case definition [35]. Local health departments must spend time and resources to follow up with providers to obtain the missing information. This has led to significant variability in state-level Lyme disease case reporting, which can affect interpretation of disease trends.

Underreporting is a known limitation of Lyme disease surveillance, but other limitations include the data’s timeliness and level of detail. Whereas the Johns Hopkins COVID-19 dashboard scrapes data from various websites in real-time [36], our dashboard does not have access to real-time data for tickborne diseases in the US and Canada. Although CDC publishes weekly provisional case counts for some tickborne diseases, Lyme disease, by far the most common tick-borne disease, is excluded from weekly reporting. CDC only publishes final annual case counts “when the year is over and all states and territories have verified their data, typically in the fall of the following year” [37]. Perhaps due to the strain that the COVID-19 pandemic has placed on health departments, CDC did not publish 2019 data until mid-2021. Although CDC supplements its annual county-level Lyme disease counts with national statistics on the seasonal distribution of cases, there is limited information on how the seasonality of Lyme disease may vary by geographic location, and how it may be changing. Ideally, we want to display county-level variation in seasonal and demographic features of Lyme disease on the “Lyme Map” application, but such data are not made publicly available.

The limitations of Lyme disease surveillance data in particular make it difficult to detect the leading edge of its geographic expansion in near real-time and hinder effective public health responses. An important and ongoing part of our initiative has been to pursue alternative and novel sources of epidemiological data. That is why, for example, we map real-time Google Trends data on the dashboard, since these data have been shown to approximate trends in the epidemiology of Lyme disease and be useful for supplementing traditional surveillance data [38]. We are also pursuing data sharing partnerships to obtain non-public data, such as health records, insurance claims, and human and veterinary laboratory testing data. Through partnerships, we hope to add data that better capture the epidemiology of Lyme and other tickborne diseases and update the data on a more real-time basis. While we recognize that estimating the true incidence of tickborne diseases is challenging, and that no single data set can capture their burden to society, we aim to push the status quo and improve the disease surveillance landscape. Adding a new section to the dashboard that features timely and unique data sets will enable the dashboard to attract more attention and become a go-to resource that amplifies the need to address the public health threat of Lyme and other tickborne diseases.

### Design trade-offs

Some design trade-offs are reflected in the current version of the dashboard. With R Shiny, performance optimization is crucial for building fast applications. Data size is one factor that can affect application speed. By design, we display most of our US data by county in order to show small-scale geographic variation, but the trade-off is that R Shiny takes longer to load a large number of geometries (approximately 3,000 counties). Improving the loading speed of the two R Shiny applications is a priority for future updates to the dashboard, especially as we prepare to add global data.

The trade-off between simplicity and complexity is also key when designing dashboards. For example, traditional dashboard layouts like the one used in the “Lyme Map” application can support numerous data visualizations, but it is important to avoid data overload and provide the right amount of information for the intended user. In designing the “Lyme Map” application for an average user, we opted to focus on the map and keep other visualizations to a minimum. We also opted for a relatively simple user interface for the “Data Explorer” application despite our initial plans to incorporate more interactive features, namely the option to filter data before download. Incorporating this feature would have required either a more complex user interface or a separate data download page, and ultimately, we wanted the main feature of the application to be data display rather than data management. With that being said, in developing future versions of the dashboard, our design choices increasingly will be informed by user feedback.

The dashboard is a novel and important contribution, one that will continue to evolve after the initial launch through the incorporation of additional data sets, geographic regions, and resources, as well as user feedback. We envision the dashboard becoming a global tracker of tickborne diseases, which will not only help to communicate to different users worldwide (e.g., public health, advocacy, donor, and funding organizations) the increasing global impact of these diseases but also serve as a resource to researchers who study their spatial variation. We also envision the dashboard becoming a hub for research collaboration and communication of geospatial findings. Lastly, we hope that in mapping reported case data and calling attention to limitations such as underreporting, our dashboard contributes to bringing about improved case reporting and a more accurate picture of the burden of Lyme and other tickborne diseases.
